# Over-expression of *GhACTIN1* under the control of *GhSCFP* promoter improves cotton fiber and yield

**DOI:** 10.1038/s41598-023-45782-0

**Published:** 2023-10-26

**Authors:** Adnan Iqbal, Sibgha Aslam, Sidra Akhtar, Qurban Ali, Abdul Qayyum Rao, Tayyab Husnain

**Affiliations:** 1 Centre of Excellence in Molecular Biology, University of Punjab, 87 West Canal Road, Lahore, 53700 Pakistan; 2 Plant Breeding and Acclimatization Institute—National Research Institute, Radzikow, 05-870 Blonie, Poland; 3 Department of Plant Breeding and Genetics, University of the Punjab, Lahore, Pakistan

**Keywords:** Biotechnology, Plant biotechnology

## Abstract

Actin dynamics is pivotal in controlling cotton fiber elongation and the onset of secondary wall biosynthesis. We report that overexpression of *GhACTIN1* under fiber fiber-specific promoter, *GhSCFP*, improves cotton fiber length, strength, and micronaire value. However, the effect of transgene has a more positive effect on fiber strength and micronaire value than fiber length. F-actin quantification and cellulose contents measurement in transgenic developing cotton fiber during the elongation phase showed an increase of up to 8.7% and 4.7% respectively. Additionally, physiological factors such as water use efficiency showed no significant change in transgenic cotton lines, while stomatal conductance and photosynthetic rate were significantly increased. Moreover, agronomical data determined that lint percentage (GOT) and seed cotton yield also increased up to 4.6% and 29.5% respectively, in transgenic cotton lines compared to the control lines. Our data demonstrate that the *GhACTIN1* gene is a strong candidate gene for cotton fiber and yield improvement.

## Introduction

Cotton fiber development largely depends on cell wall biosynthesis and cytoskeleton arrangement. Cytoskeleton dynamics control many cellular processes, such as the movement of organelles, cell wall formation, and cell division. Microfilament (actin-filament), microtubules, and intermediate filaments are the main constituents of the cytoskeleton^1^. In most cells, the actin filaments are involved in secretory vesicle transportation to the cell membrane and cell wall, enhancing cell expansion. Tip growth and cell elongation are also regulated by the actin cytoskeleton^2^. In plants, actin proteins are expressed by dozens of *ACTIN* genes family, while cotton plants have been identified with 16 *ACTIN* genes^3^. Actin is expressed in a monomeric form which is known as G-actin. The G-actin polymerizes to form a filament known as F-actin^4^. Formation of actin filaments by monomeric actin includes nucleation, polymerization/capping, and F-actin bundling & cross-linking activities. Many actin-binding proteins (ABPs) are divided according to their association among G-Actin binding/ G-actin capping proteins, F-Actin regulators, or actin-binding proteins (ABPs), which are involved in either polymerization or depolymerization and proteins that serve to crosslink and/or bundle the actin microfilaments^5^.

ADF (actin depolymerization factor) and profiling are important ABPs^6,7^. Previous studies have validated that profiling, such as *GhPFN2* and *ADF* regulates actin dynamics by Ca^2^^+^ stimulation^7^. Annexins are a multigene family and are categorized as ABPs. Huang et al.^8^ revealed that cotton annexin anxGb6 interacts with fiber *GACTIN1*, fiber-specific actin, and plays an important role in fiber elongation. Li et al.^3^ have reported that *GhACTIN1* was expressed predominantly during cotton fiber elongation. Furthermore, actin-turnover during fiber development is vital to keep the process uninterrupted. RNAi inhibition of *GhACTIN1* in cotton fiber drastically reduced the F-actin filaments network consequently, fiber length and strength were found to be reduced, which suggested that the *GhACTIN1* gene has a major role in fiber elongation, but the contribution of other genes, such as *GhACTIN2* and *GhACTIN5* cannot be completely ruled-out^3^.

Cotton fiber provides a good model for studying cell elongation and cell wall biosynthesis using biotechnological approaches^9,11^. Improved fiber yield and quality can be achieved through genetic modification. The idea of over-expression of a certain gene to achieve the preferably required characteristic has become widespread such as fiber elongation has been reported by Zhang et al.^10^ through over-expression of *GhFIM-2.* FIM (Fimbrin) are actin-bundling proteins vital in pollen-tube growth in lily and Arabidopsis^11,12^. Over-expression of *GhFIM-2* from the FIM family enhances the actin filament bundling at the fiber elongation stage and helps in propelling the secondary wall biosynthesis. Thus, indicates the role of *GhFIM-2* in fiber development by actin dynamic re-arrangement^10^. Over-expression of *GhPFN-2*, a profilin, in cotton fibers results in secondary cell wall synthesis initiation by terminating the elongation phase before the time. This early termination of the elongation phase and early onset of secondary wall synthesis resulted in the short length of cotton fibers compared to the wild type.^13^. Over-expression of *AKR2A* (ankyrin repeat-containing protein 2A), an Arabidopsis gene, in cotton plants revealed that it promotes the elongation of cotton fiber by increasing the VLCFA contents in transgenic lines compared to non-transgenic. *AKR2A* gene also promotes fiber elongation by signaling of hydrogen-peroxide^16^.

The expression of a transgene in cotton fiber requires strong fiber-specific promoters to ensure improved yield and quality. However, limited investigations have been made on fiber-specific promoters. To explore the molecular basis of cotton fiber development Hou et al.^14^ reported that *GhSCFP* (*Gossypium hirsutum* seed coat and fiber-specific protease) expression was higher during fiber initiation and elongation. With this background knowledge, the current study was designed to improve cotton yield and fiber quality by over-expression of *GhACTIN1* under fiber-specific promoter *GhSCFP* in local cotton cultivars.

In our previous study, we showed how *GhWLIM5* helps in fiber strength improvement by interacting with actin, and we proposed the role of the *GhACTIN1* gene in fiber improvement^18^. The current study was designed to characterize the *GhACTIN1* gene by transforming the local cotton variety CEMB-88. The over-expression of *GhACTIN1* improved the cotton fiber length, strength, and fineness and improved cotton yield.

## Materials and methods

### Cloning of the GhACTIN1 gene

The fasta sequence of the *GhACTIN1* gene (AY305723.1) was retrieved from the National Center for Biotechnology Information (NCBI) and was submitted to the IDT database for codon-optimization (https://www.idtdna.com/pages/tools/codon-optimization-tool) by replacing less frequently used codon by favorably expressed codon in cotton plant^15^. The codon-optimized sequence of *GhACTIN1* gene under fiber-specific promoter, seed coat, and fiber-specific protease (SCFP), (GQ411495.1), with *Pst I* and *Sac I* restriction sites, was synthesized in pUC57 vector with ampicillin as selection marker on a commercial basis. The pUC57_*GhACTIN1* plasmid was transformed in *Top10* competent cells and the isolated plasmid was confirmed through restriction digestion analysis using FastDigest enzymes *Pst I* and *Sac I.* The enzyme-restricted gene was ligated into the pCAMBIA-1301 vector using Rapid DNA Ligation Kit (Thermos Fisher Scientific Cat#K1423) to make the pCAMBIA1301_*GhACTIN1* construct. After the confirmation of compactness and successful ligation through restriction digestion and PCR-based amplification of the gene pCAMBIA1301_*GhACTIN1* construct was transformed into *Agrobacterium* through electroporation. PCR-based confirmed *Agrobacterium* colonies were further used for plant transformation. It has been confirmed that the experimental data collection, complied with relevant institutional, national, and international guidelines and legislation with appropriate permissions from authorities of the Centre of Excellence in Molecular Biology, University of the Punjab, Lahore, Pakistan.

### Cotton (Gossypium hirsutum) transformation

Local cotton variety CEMB-88 was selected for the transformation of the *GhACTIN1* gene. The cotton variety CEMB-88 was selected based on its best germination rate reported previously by Iqbal et al.^18^. The cotton seeds were acquired from Multan CEMB Research Farm, situated in the southern part of Punjab, Pakistan, and subjected to the *Agrobacterium*-mediated shoot apex cut method of plant transformation method reported by Iqbal et al. and *Rao *et al*.*^16,18^.

### Molecular analyses of transgenic cotton plants

#### Polymerase chain reaction (PCR)

Genomic DNA was extracted from young leaves of both transgenic and non-transgenic control cotton plants by using the CTAB method as was done by Horne et al.^17^, and screening of transgenic cotton plants was done through amplification by using PCR. The genomic DNA of putative transgenic cotton plants and non-transgenic cotton plants was used as a DNA template. The primers were designed by considering the promoter for forward and the *GhACTIN1* sequence for reverse primer (Act-F 5’_ GATAATGGTACTGGTATGGTGAAAG_3’ & ACT-R 5’_ GTTGTAAACATGTATCCTCTCTCAG_3’).

#### GUS (Histochemical) assay

Transient GUS assay of cotton fiber was determined to confirm the successful functioning of gene cassette as was determined by Satyavathi et al.^18^. Briefly: GUS solution (25 mg/L X-gluc, 10 mM EDTA, 100 mM NaH2PO4, 0.1% Triton X-100 and 50% methanol, pH 8.0) was prepared and avoided from light. Fibers of transgenic cotton plants were immersed in GUS solution, incubated overnight at 37 °C, and observed with the help of a microscope for the appearance of blue color.

#### Quantitative real-time PCR (qRT-PCR)

RNA was extracted from cotton fiber using the method reported by Iqbal et al.^18^ and reverse transcribed chemically into cDNA using cDNA Synthesis Kit (Fermentas, cat#1622). Expression analysis of the *GhACTIN1* gene at the mRNA level was done through quantitative real-time PCR in transgenic and non-transgenic control cotton fiber. The relative quantification of gene expression was done using (BIO-RAD) iQ5 Cycler. Data normalization was done using GAPDH as internal control and non-transgenic/wild-type cotton plants as negative control. All samples were analyzed in triplicate with the primers: Act-F 5’-GGCAGATGGTGAGGCTATTC-3’ & Act-R 5’-CTTGCTTTGGGCTTCATCTC-3’. After completing Rt-qPCR, the analyses for relative gene expression were performed by Qiagen tool, Relative Expression Software Tool REST abbreviated as REST (http://rest.gene-quantification.info/) and ANOVA (Analysis of variance) was performed to compare the transgenic and non-transgenic control cotton plants for expression of the transgene.

### Biochemical and physiological analyses of plants

#### Quantification of F-actin

For total protein extraction from cotton fibers by following the method with some modifications reported by Dure & Chlan^19^. Cotton fibers were carefully removed from 16DPA ovules. The dried weight of fiber was taken in a 1.5 mL tube with an addition of 5 parts of insoluble PVPP (Polyclar, AT) to each part of the dry weight of fiber (w/w); 15 mL of extraction buffer (50 mM Tris–HCl, pH 8 to 6; 2% 2-mercaptoethanol; 2% SDS) for each 100 mg of dried sample was added and homogenized. After homogenization, the mixture was incubated at 100 °C in a dry heat bath for 5 min and subjected to centrifugation to separate pellet, cell debris, and PVPP. The supernatant was shifted to the new 1.5 mL tubes, and 10 volumes of ice-chilled acetone were added and placed overnight at -20 °C for protein precipitation. The precipitated protein pellet was obtained through centrifugation of the sample mixture. The acetone supernatant obtained was discarded and re-suspended in PBS. The F-actin of the total protein was stained using FITC-phalloidin molecular probes^20^. The Fluorescence intensity of FITC-phalloidin-stained F-actin was recorded by spectrophotometer (Thermo Scientific Spectronic 200E) at 530 nm wavelength.

#### Cellulose contents measurement

100 mg fiber samples were treated with 80% hot acetic:nitric (10:1) reagent for 1 h. The samples were washed thrice with distilled H_2_O and a final wash with absolute ethanol before air drying. The final and the initial weight ratio of the sample residues were taken as cellulose contents as was done by^21^.

#### Measurement of stomatal conductance, photosynthetic rate, water use efficiency and transpiration rate

Photosynthetic rate, water use efficiency, and evaporation rate were measured using a CIRAS-3 portable Infrared Gas Analyser (PP Systems Amesbury, USA) according to the settings described in the CIRAS-3 user manual. The apparatus settings were adjusted ambient for light, CO_2,_ H_2_O, and temperature and the chamber for leaf area was fixed at 4cm^2^^.^ The recordings were made in triplicate for each plant.

#### Cotton fiber analyses

The transgenic and non-transgenic cotton fiber was subjected to analyses including fiber length, strength, micronaire value maturity, and uniformity index. High Volume Instrument SW v3.3.5.57 was used to perform these fiber quality tests at the fiber quality domain of Central Cotton Research Institute, Multan, Pakistan (http://www.ccri.gov.pk/). The samples were taken in triplicate biological control, and the mean values have been represented in the data.

### Microscopic analyses of fiber

#### Scanning electron microscopy

Dried mature cotton fibers from transgenic and non-transgenic cotton fibers were excised, and the middle section of fiber was analyzed under a scanning electron microscope (M-SU8010, Hitachi Japan). The screw-pitch and the distance of fiber rotation in "360°" rotation were measured thrice for every sample using × 400 and × 4000 power lenses as performed by^22^. The samples were taken in triplicate biological control, and the best representative has been presented in the data.

#### Fluorescence in-situ hybridization (FISH) analyses

FISH analysis was performed by following Mahmood-ur-Rahman et al.^23^ method. The probes were labelled using Label IT FISH Cy3 kit by MIRUS (Cat#6512). Germinated cotton seeds were used for slide preparation, and prepared slides were hybridized with probes following the chromosomal staining with DAPI. A fluorescent microscope (Zeiss AX10) was used to detect the fluorescent signals using blue and red filters for DAPI and PI respectively. Karyotyping of transgenic and non-transgenic control cotton chromosomes was done using Genus Software Inc. provided by Imaging Cytovision Systems. The position of transgene integration and copy number was determined by direct visualization of fluorescent signals on chromosomes of transgenic cotton plants at metaphase.

### Determination of agronomical data

#### Seed index

The seed index was calculated by ginning seeds of both transgenic and non-transgenic cotton lines. The hundred disease-free cotton seeds were selected and weighed in grams (gs) on an electrical scale and considered as seed index.

#### Lint % (GOT)

Lint % is usually referred to as ginning out turn. For measuring ginning out turn, the dry and clean cotton seeds, picked from the cotton bolls, were weighed in grams (g) and subjected to ginning for weighing ginned lint in grams (g). The given formula was used for calculating lint percentage (GOT).

$$ {\text{Lint}}\% \left( {{\text{GOT}}} \right) = \frac{{{\text{Weight}}\;{\text{of}}\;{\text{lint}}}}{{{\text{Weight}}\;{\text{of}}\;{\text{Seed}}\;{\text{cotton}}}} \times 100 $$

#### Seed cotton yield

The seeds from selected transgenic and control cotton lines were collected separately. The weight was measured on the weighing balance, and each cotton line's total seed weight was divided by the total number of plants in respective lines to calculate seed cotton yield.

#### Boll weight per plant

Average dry boll weight was calculated by weighing all the mature bolls picked from transgenic and control cotton plants and by dividing them to the total number of bolls per plant. The fresh boll at weight at 25 DPA was calculated by picking ten fresh bolls from each transgenic and control cotton plant of their respective lines followed by measurement on scale and calculation of their mean values.

#### Statistical data analyses

Morphological and agronomical characteristics of transgenic cotton lines at T_1_ progeny were studied. One-way (Dunnett’s Test) and two-way (Turkey’s Test) ANOVA (Analyses of Variance) were performed for the comparison of significance level between transgenic and non-transgenic cotton lines. GraphPad Prism software (7.0 for Windows) was used to perform these statistical data analysis tests. The graphical bars with statistically significant values compared to non-transgenic control having *p-values* ≤ 0.05, 0.01, 0.001, and 0.0001 were indicated by asterisks as *, **, ***, and **** respectively, while non-significant values were denoted as ‘x’.

## Results

### Cloning of the GhACTIN1 gene

**Figure 1:**
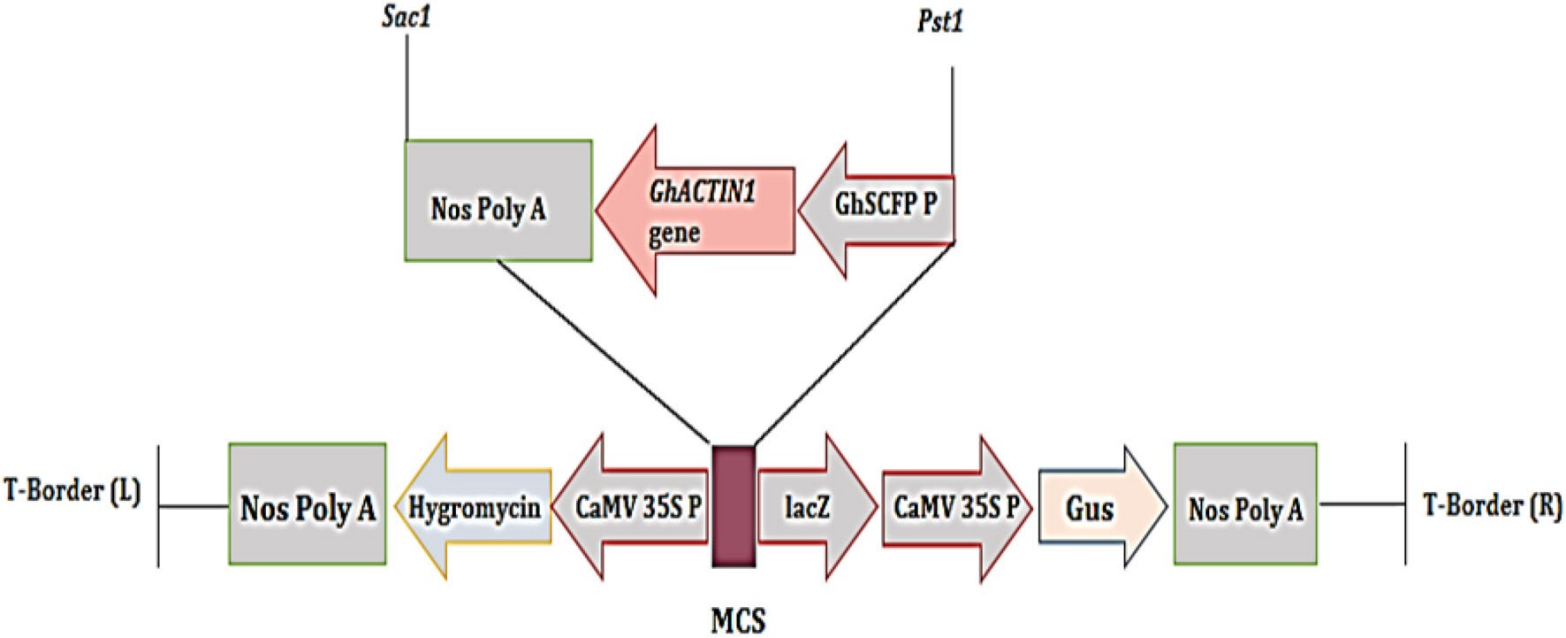
Schematic representation of *GhACTIN1* gene cassette under the control of *GhSCGP* promoter and NOS terminator in pCAMBIA-1301 vector.

The codon-optimized *GhACTIN1* cassette ligated into the pUC-57 cloning vector, was subjected to bacterial transformation using Top10 of *E. coli*. The restriction-digested band of 2 kb using *PstI* and *SacI* enzymes confirmed the ligation of the *GhACTIN1* gene cassette into the PUC-57 vector. (Supplementary material: Fig. S1). The *GhACTIN1* cassette was ligated in the pCAMBIA-1301 vector (Fig. 1). The amplification of the 577 bp fragment, resolved on 1.5% agarose gel, confirmed the successful ligation of the *GhACTIN1* cassette in the pCAMBIA-1301 vector construct (Supplementary material: Figs. S2A, S3A, S3B). The compactness of the construct was confirmed through the restriction digestion method. The excision of a 2 kb fragment, resolved at 0.8% agarose gel, confirmed the compactness of the construct pCAMBIA-1301_*GhACTIN1* (Supplementary material: Fig. S2). The successful transformation of pCAMBIA-1301_*GhACTIN1* Construct into Agrobacterium strain LBA4404 by electroporation method was confirmed by colony PCR. The amplification of the 577 bp fragment, resolved on 1.5% agarose gel, confirmed the successful introduction of pCAMBIA-1301_*GhACTIN1* into Agrobacterium strain LBA4404 (Supplementary material: Fig. S3).

### Transformation of the GhACTIN1 gene in cotton plants

**Figure 2:**
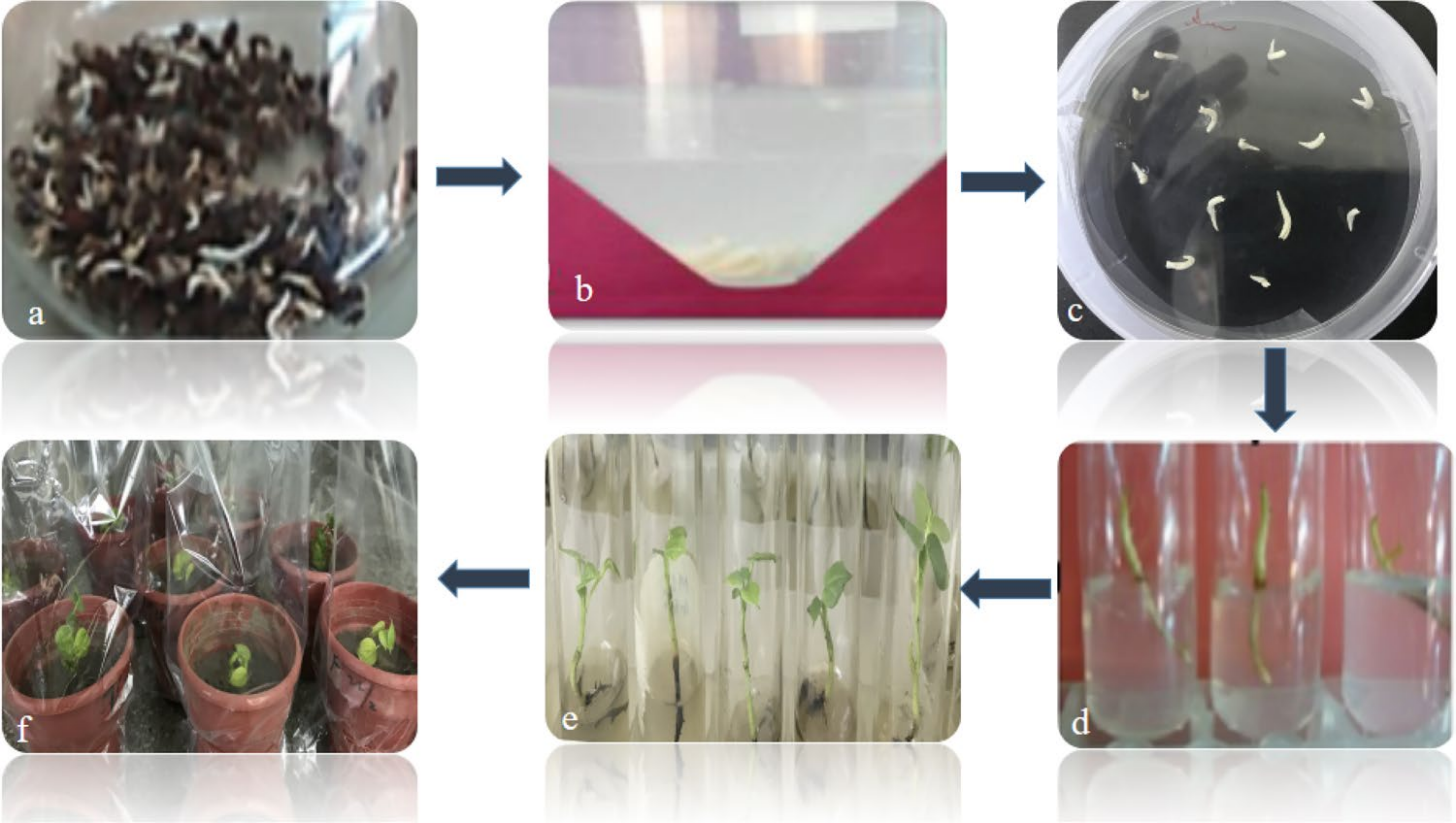
An Overview of *Agrobacterium*-mediated Cotton Transformation (**a**) sterilized germinated seed (**b**) Agrobacterium inoculum of excised cotton embryos (**c**) Co-cultivation (**d**) Roots sprouting embryos on selection medium (**e**) Establishment of plantlets in tubes (**f**) Putative transgenic cotton plants shifted to soil.

CEMB-88 variety was selected for transformation. A total of 7,500 isolated embryos from sterilized germinated cotton seeds were subjected to excision at the shoot apex region by a sharp scalpel and treated with *Agrobacterium* inoculum containing the *GhACTIN1* gene and co-cultivated on zero MS media plates. After a week, the root-sprouting embryos were shifted to culture tubes containing MS selection media. A total of 78 plants survived on the selection MS media, and the transformation efficiency was 1.04%. After 5–6 weeks, the surviving plantlets were shifted to pots containing autoclaved soil (Fig. 2a–f).

### Establishment of putative transgenic cotton plants in the field

Out of 78 putative transgenic cotton plants, shifted into pots for acclimatization, only 27 plants survived. The well-established pot plants were then shifted to the CEMB field to grow in the natural environment.

### Molecular analyses of putative transgenic cotton plants in T_***0***_ progeny

Putative transgenic cotton plants were subjected to molecular analyses such as PCR, transient expression of GUS through GUS assay, and relative expression of the *GhACTIN1* gene at mRNA level to confirm the integration and expression of the transgene in cotton plants.

### Confirmation of transgene through amplification by PCR

**Figure 3:**
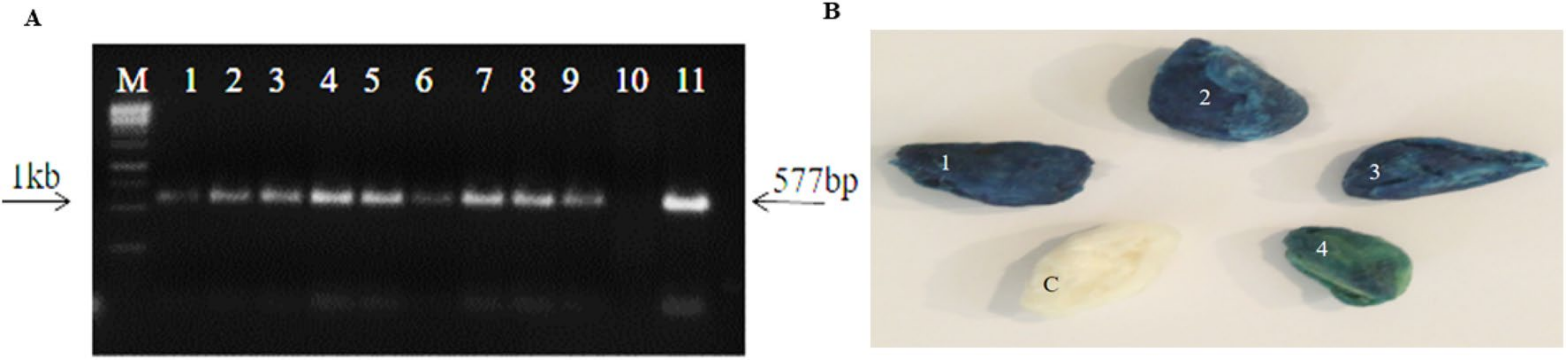
(**A**) PCR Analyses of Putative Transgenic Cotton Plants in T_0_ Progeny. M: 1 kb Ladder; Lane 1–9 Transgenic plants with amplification of 577 bp fragment; Lane 10 Negative Control (Non-transgenic); Lane 11 Positive Control plasmid (pCAMBIA-1301_*GhACTIN1* Plasmid. (**B**) GUS Staining Assay of Cotton Fibers. (1–4) Transgenic Cotton fibers with the appearance of blue color show the GUS activity. (**C**) Non-transgenic cotton fiber (negative control) without the appearance of blue color shows no GUS activity.

PCR analyses were performed using extracted genomic DNA as described in 3.7 using gene-specific primers. The amplification of the 577 bp fragment confirmed the successful introduction of the transgene in the cotton variety CEMB-88. No PCR amplification was observed in control non-transgenic cotton plants (Fig. 3A).

### Histochemical GUS assay

Histochemical GUS expression assay of young developing fiber attached to the ovules of both putative transgenic and non-transgenic control cotton plants was done by application of substrate to the cotton fibers, respectively. The appearance of blue color in transgenic cotton plant fiber confirmed the initial screening of the *GhACTIN1* gene in *Agrobacterium*-inoculated cotton plants for their successful introduction and expression of the cassette in fibers. However, no GUS activity (appearance of blue color) was observed in non-transgenic cotton plant fibers (Fig. 3B).

### Development of advanced generation of GhACTIN1 transgenic cotton plants

The seeds from the transgenic cotton plants (A-08, A-15, A-24, and A-36) analyzed by qRT-PCR (Supplementary material: Fig. S4) were raised to their T_1_ generation in the form of four respective lines. Each transgenic cotton line was comprised of 7 plants. Non-transgenic cotton plants were also raised in a separate line as a control line to study molecular, biochemical, and physiological characteristics comparatively.

### Molecular analyses of T_***1***_ generation of transgenic cotton plants

#### Confirmation of advanced generation of transgenic cotton lines through PCR amplification

All four transgenic cotton lines' advanced-generation cotton plants were subjected to PCR amplification by using gene-specific primers and isolated DNA as a template. The amplification of the 577 bp band resolved on 0.8% agarose gel in T_1_ transgenic cotton plants confirmed the successfully integrated transgene after being segregated in the advanced generation of cotton. However, no amplification of the DNA band was observed in the non-transgenic control cotton line (Supplementary material: Fig. S5).

#### Quantitative real-time PCR (qRT-PCR) of transgenic cotton plants (T_*1*_ Generation)

**Figure 4:**
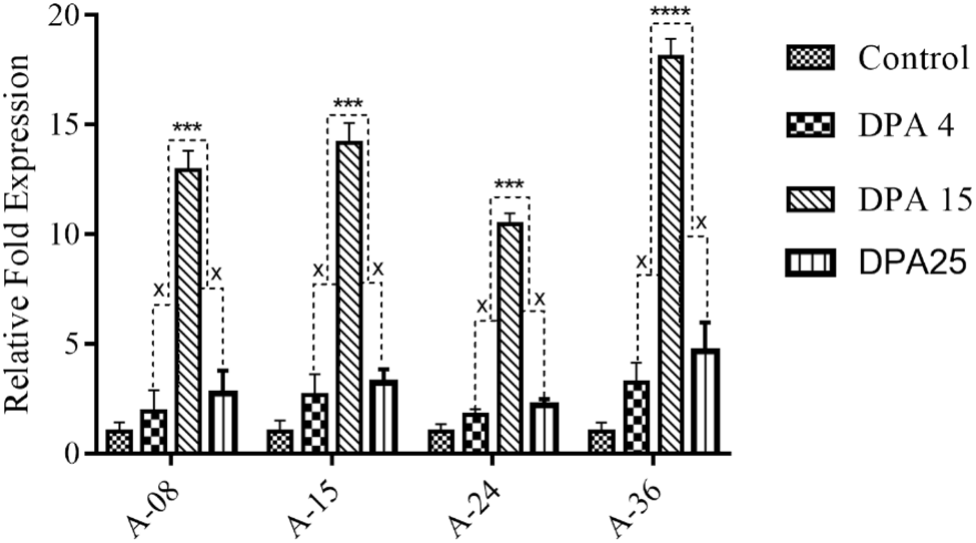
Relative Fold Expression at different Fiber developmental phases (Initiation, Elongation, and Secondary Wall Synthesis). Each bar is the mean value representation of three replicates and Two-way ANOVA was performed for statistical analysis.

To quantify the *GhACTIN1* gene mRNA transcript level, total mRNA was isolated from different developmental stages of cotton fiber such as initiation (4DPA), elongation (15DPA), along with secondary wall synthesis (25DPA), and reverse transcribed into cDNA. Exponential amplification of fiber cDNAs through real-time PCR revealed that *GhACTIN1* gene mRNA level was very low at the end of initiation or the beginning of elongation (4DPA) and was maximum during elongation (15DPA) while gradually decreased at the end of elongation or the beginning of secondary wall synthesis (25DPA). The transgenic line A-36 showed a maximum increase in mRNA expression of *GhACTIN1* during the fiber elongation phase, which was calculated to be 18.09 folds, while during initiation and secondary wall synthesis, it was estimated to be 3.2 and 4.6 folds, respectively when compared to the non-transgenic control line. The mRNA expression patterns were recorded in other transgenic cotton lines. Statistical analysis, two-way ANOVA, of the group data, indicated a significant difference in transgene mRNA expression during elongation time compared to initiation and secondary wall synthesis time (Fig. 4).

### Determination of Transgene Integration Location and Copy Numbers at Chromosomal Level of Transgenic Cotton Plants

#### FISH (Fluorescent in situ hybridization) analysis

**Figure 5:**
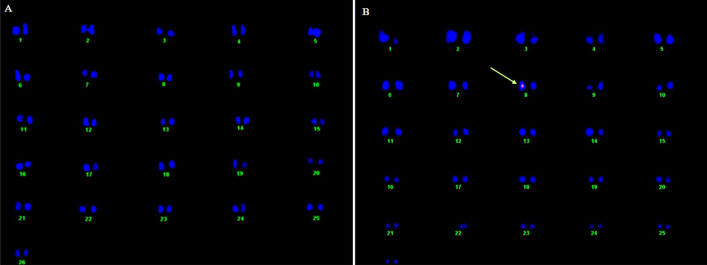
Karyogram indicating integration and location of *GhACTIN1* gene in the cotton genome. (**A**) Non-transgenic control plant with no fluorescent signal (**B**) Arrow indicates fluorescent signal at chromosome 8 of transgenic plant in a hemizygous form.

*GhACTIN1* gene integration location on chromosome and transgene copy numbers in transgenic cotton lines was determined through *Fluorescent *In Situ* Hybridization* in advanced generation (T_2_). Transgenic cotton line A-36, which showed the maximum improvement in fiber characteristics, including fiber strength, length, micronaire, and maturity ratio along with higher expression of the *GhACTIN1* gene was selected for *Fluorescent *In Situ* Hybridization* analysis. The FISH analysis revealed that the *GhACTIN1* gene-specific probe hybridized at chromosome number 8 in hemizygous form (Fig. 5B). The single bright fluorescent signal on chromosome number 8 indicates a single copy number in transgenic cotton line A-36 while FISH analysis of the non-transgenic control line determined no fluorescent signal (Fig. 5A).

### Plant biochemical and physiological analyses

#### Quantification of F-actin

**Figure 6:**
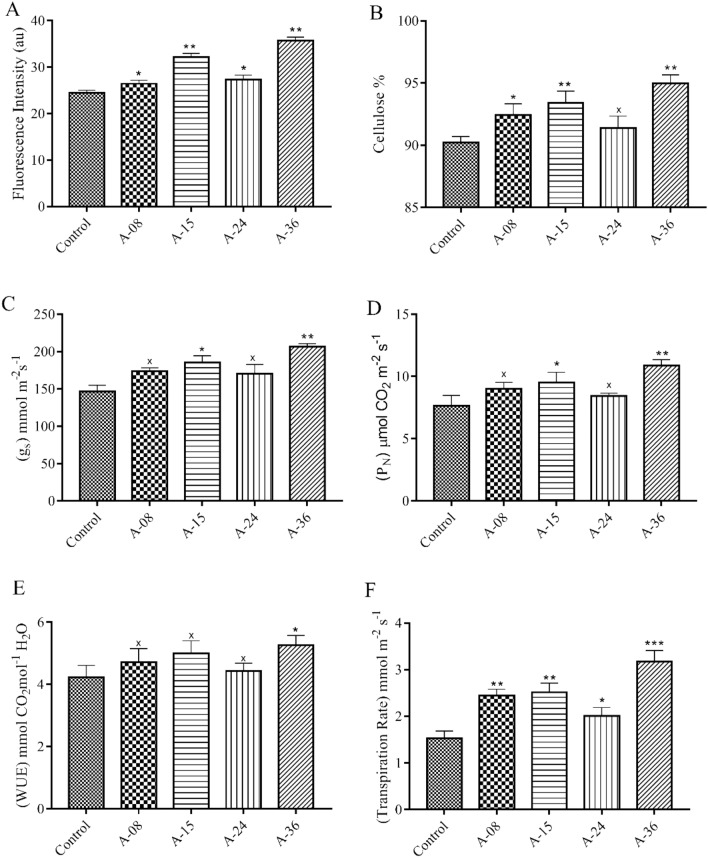
Biochemical and Physiological Analysis: (**A**) F-Actin Filament Quantification in Elongation Phase at 16DPA (**B**) Cellulose Contents Measurement in Mature Fibers of Transgenic and Control Plants (**C–F**) Measurement of Stomatal Conductance, Photosynthetic Rate, Water Use Efficiency and Transpiration Rate. Each bar is the mean value representation of three replicates and One-way ANOVA was performed for statistical analysis.

F-actin filament quantification analyses of 16DPA cotton fibers of transgenic cotton lines compared to the non-transgenic control line revealed a significant increase in the quantity of F-actin filament. Transgenic lines A-08, A-15, A-24, and A-36 showed fluorescence intensity of 32.1, 26.4, 27.3, and 35.7au with increments of 7.8%, 6.4%, 6.6%, and 8.7%, respectively, when compared to 24.4au fluorescence intensity of non-transgenic control cotton fiber (Fig. 6A).

#### Measurement of cellulose contents

Cellulose contents comparative analyses result of the transgenic and control cotton lines showed higher values in transgenic cotton lines than control. A maximum of 4.7% increment in cellulose contents was observed in transgenic cotton line A-36 while A-08, A-15, and A-24 showed an increment of 2.2%, 3.2%, and 1.3% compared to non-transgenic cotton fibers (Fig. 6B).

#### Stomatal conductance

Stomatal conductance measures CO_2_ absorption rate with the evaporation of H_2_O through the stomatal aperture. The stomatal conductance of transgenic cotton lines A-08, A-15, A-24, and A-36 was calculated to be 173.6, 185.3, 170.2, and 206.5 mmol m^-2^ s^-1^ values correspondingly compared to non-transgenic control line 146.6 mmol m^-2^ s^-1^ (Fig. 6C). One-way ANOVA analysis indicates that transgenic lines A-15 and A-36 significantly differed from the non-transgenic control line in stomatal conductance.

#### Photosynthetic rate measurement

The photosynthetic rate (P_N_) of transgenic and non-transgenic control plants was measured through IRGA CIRUS3. The photosynthetic rate was 9.0, 9.5, 8.4, and 10.8 µmol CO_2_ m^-2^ s^-1^ for transgenic lines A-08, A-15, A-24 and A-36 while 7.6 µmol CO_2_ m^-2^ s^-1^ for non-transgenic control cotton plants. The photosynthetic rate in cotton plant lines A-15 and A-36 was significantly higher in P_N_ values than non-transgenic control cotton plant line when analyzed through ANOVA (Fig. 6D).

#### Water use efficiency

The WUE of transgenic cotton lines A-08, A-15, A-24, and A-36 was found to be 4.7, 5.0, 4.3, and 5.2 mmolCO_2_ mol^-1^ H_2_O, respectively when compared to non-transgenic control cotton line with 4.2 mmol CO_2_ mol^-1^ H_2_O. No significant difference in water use efficiency was observed except in transgenic cotton line A-36 (Fig. 6E).

#### Rate of transpiration

A positive correlation between transpiration rate and photosynthetic rate (CO_2_ assimilation rate) was observed in transgenic cotton lines. Transpiration rate in cotton lines A-08, A-15, A-24, and A-36 was found to be 2.4, 2.5, 2.0, and 3.18 mmol m^−2^ s^-1^ in a sequential order compared to the control line where it is recorded to be 1.5 mmol m^−2^ s^-1^. A 0.5 to 1.68 mmol m^−2^ s^−1^ increment in transpiration rate was recorded (Fig. 6F).

### Determination of cotton fiber quality and its microscopic examination

#### Cotton fiber length

**Figure 7:**
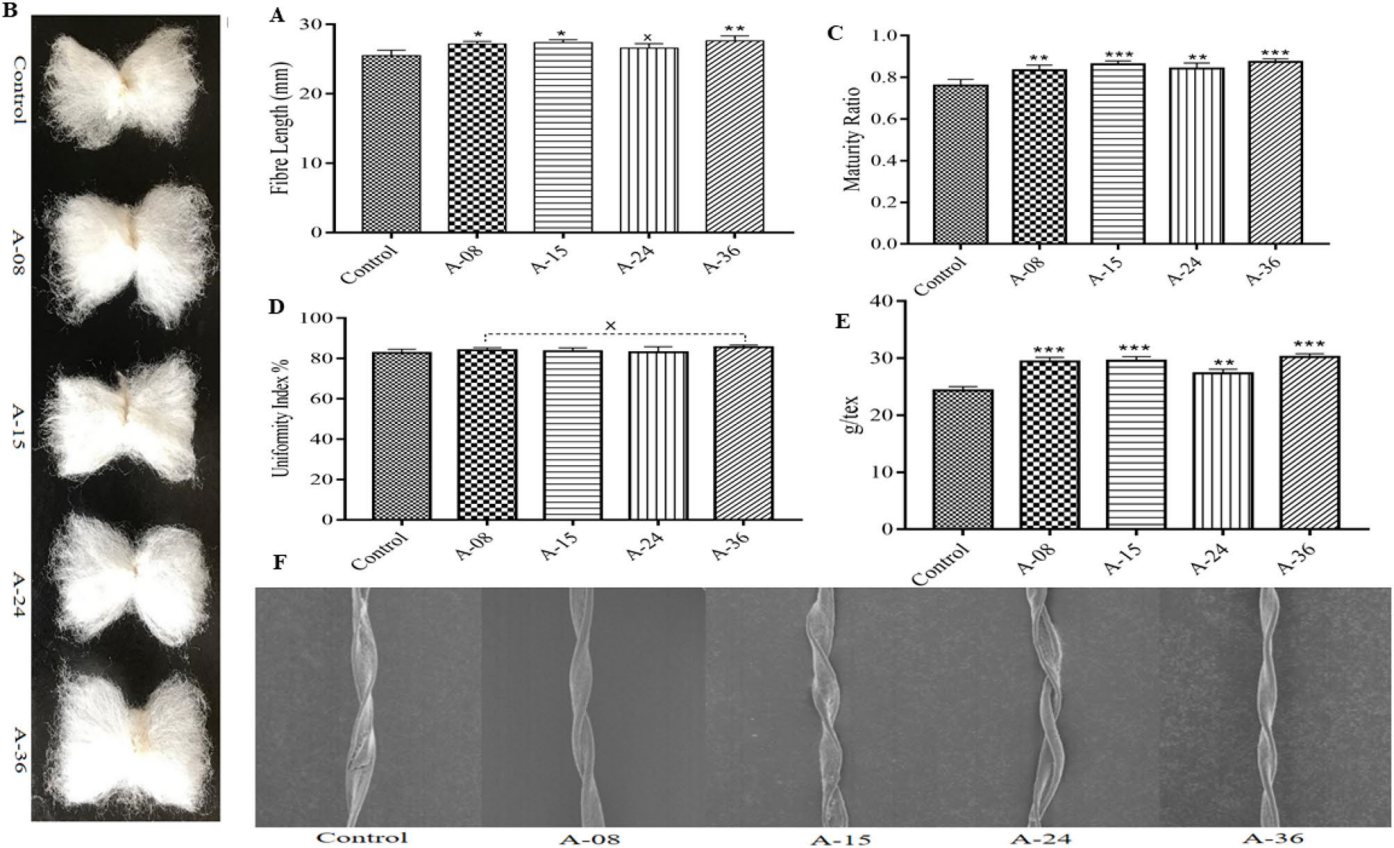
Fiber qualitative and SEM analyses. (**A**) Represents the comparison of cotton fiber lengths (mm) of transgenic and non-transgenic control plants (**B**) Photographic representation of fiber. (**C**) Comparison of improved maturity ratio in transgenic cotton fiber compared to non-transgenic control (**D**) Representation of uniformity index of cotton fiber in transgenic and non-transgenic control lines (**E**) Represents the increased fiber strength (g/tex) of transgenic cotton fiber compared to non-transgenic (**F**) SEM images showing an increased number of twists per unit length of transgenic cotton fiber compared to non-transgenic control (Each bar is the mean value representation of three replicates and One-way ANOVA was performed for statistical analysis).

Fiber length is one of the most significant quantitative traits from the commercial point of view. The High-Volume Instrument analysis of cotton fiber of transgenic cotton lines A-08, A-15, A-24, and A-36 showed the lengths as 27.1, 27.3, 26.6, and 27.6 mm compared to 26.2 mm of non-transgenic control cotton line. Transgenic cotton lines A-08, A-15, and A-36 significantly increased fiber length, while transgenic cotton line A-24 showed a constant value with no significant impact on fiber length in contrast to the non-transgenic control cotton line. A maximum of 5.3% increment in the transgenic cotton line was recorded compared to the non-transgenic control cotton (Fig. 7A,B).

#### Cotton fiber strength

Cotton fiber strength is one of the important traits among fiber quality determination parameters for the textile industry as the strength of fiber further affects neps production and the spinning performance. The fiber of the selected transgenic cotton line along with the non-transgenic control line analyzed by CCRI, labs revealed that the strength of transgenic cotton lines namely A-08, A-15, A-24, and A-36 was determined to be 27.3, 29.5, 29.4 and 30.2 g/tex sequentially in comparison to the non-transgenic control cotton line which was 24.3 g/tex. When the data was statistically evaluated, all transgenic cotton lines showed a significant increase in fiber strength compared to the non-transgenic control line. Overall, a maximum of 24.2% increment in fiber strength of transgenic cotton fiber was observed (Fig. 7E). Scanning electron microscopic analysis, zoomed at × 400 of the transgenic cotton fiber showed a higher number of twists per unit area compared to non-transgenic control cotton fiber. The higher number of twists can directly be correlated with the higher strength of cotton fiber (Fig. 7E,F).

#### Maturity ratio and uniformity index of cotton fiber

Fiber maturity is a ratio of cell wall thickness to the diameter or the cell wall thickness compared to the size of the lumen, and its values of 0.7 to 0.9 are considered to be optimum. Similarly, the uniformity index (UI%) is the ratio of mean length to the UHML (upper half mean length). The maturity ratio of transgenic cotton lines, namely A-08, A-15, A-24, and A-36 was found to be 0.83, 0.86, 0.84, and 0.87, respectively, relative to the maturity ratio of non-transgenic control cotton line, which was recorded to be 0.76. Statistically, all the transgenic cotton lines showed significant improvement in maturity ratio compared to the non-transgenic control cotton line. A maximum increase of 10.5% was observed in the maturity ratio of transgenic cotton lines (Fig. 7C). However, no significant difference was obtained in the uniformity index of transgenic cotton lines when compared with non-transgenic control cotton lines. The uniformity index of transgenic cotton lines A-08, A-15, A-24, and A-36 was recorded to be 84, 83.5, 83.1 and 85.6%, respectively compared to 82.7% of the non-transgenic control line (Fig. 7C,D).

#### Micronaire values of cotton fiber

**Figure 8:**
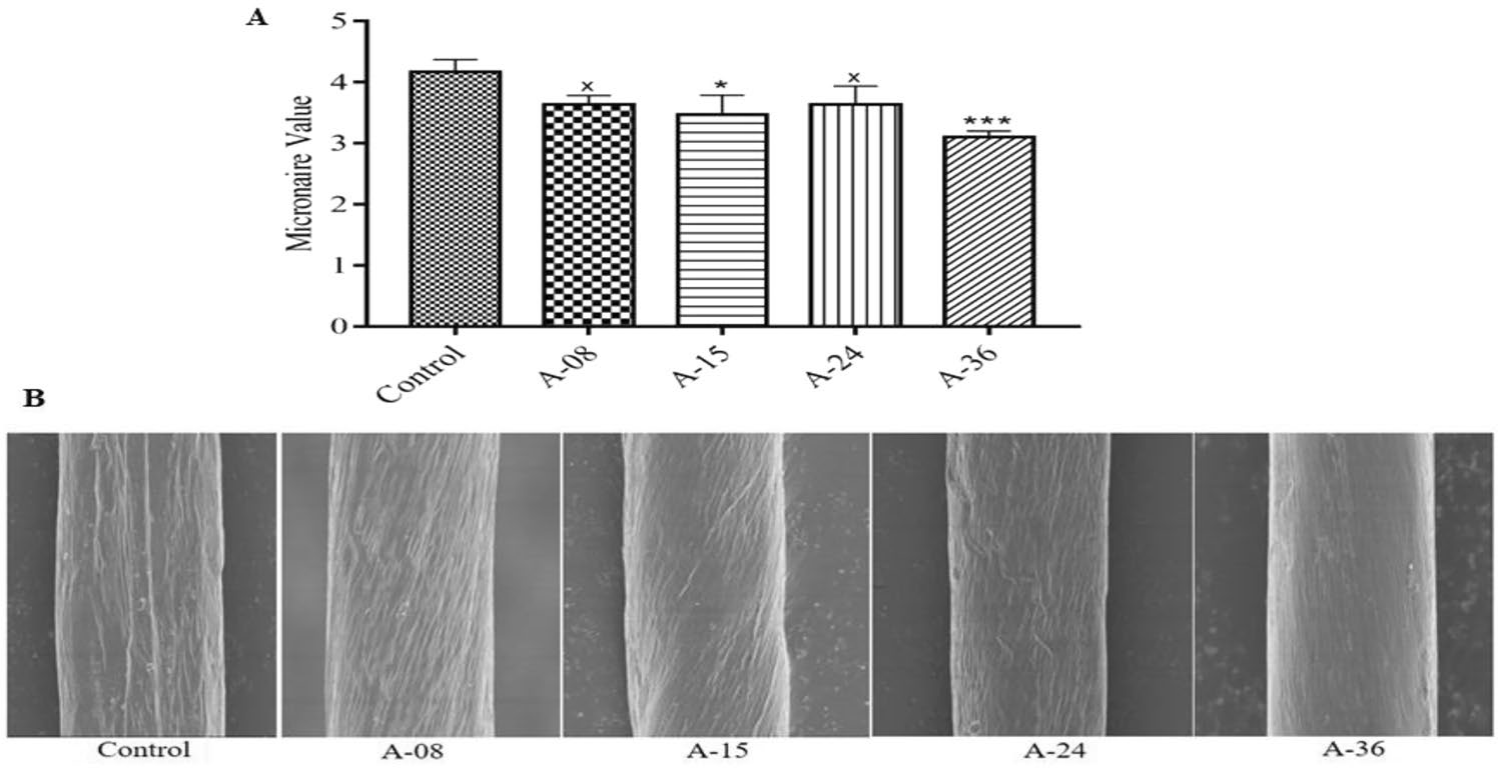
Fiber micronaire and SEM analysis. (**A**) Represents the improved micronaire values of transgenic cotton fibers compared to non-transgenic control (Each bar is the mean value representation of three replicates and One-way ANOVA was performed for statistical analysis) (**B**) SEM images with improved fineness of transgenic cotton fiber.

Micronaire is defined as the combination of fiber fineness and maturity. The lower the micronaire values, the better the fiber fineness and maturity ratio. The fiber samples from four transgenic cotton lines along with non-transgenic control line samples were subjected to the air-flow resistance measuring method at CCRI lab, and maicronaire values calculated to be 3.6, 3.4, 3.6, and 3.1 of A-08, A-15, A-24, and A-36 transgenic cotton lines respectively in comparison to 4.1 of the non-transgenic control line. Transgenic cotton line A-36 showed a maximum value of 24.3% (Fig. 8A). Further scanning electron microscopic analysis of transgenic and control cotton fiber, observed at × 4000 revealed that the smoothness of the transgenic cotton fiber surface as compared to the non-transgenic control line, which is directly proportional to the cotton fiber fineness (Fig. 8B).

### Agronomical characteristics of transgenic and non-transgenic cotton plants in T_2_ generation

Agronomical characteristics of transgenic cotton plants compared to the non-transgenic control line were taken into account to define any comparable change in both group of plants which may be attributed to insertional change or any contribution from genetic modification.

#### Seed index, ginning out turn (GOT), and seed cotton yield

**Figure 9:**
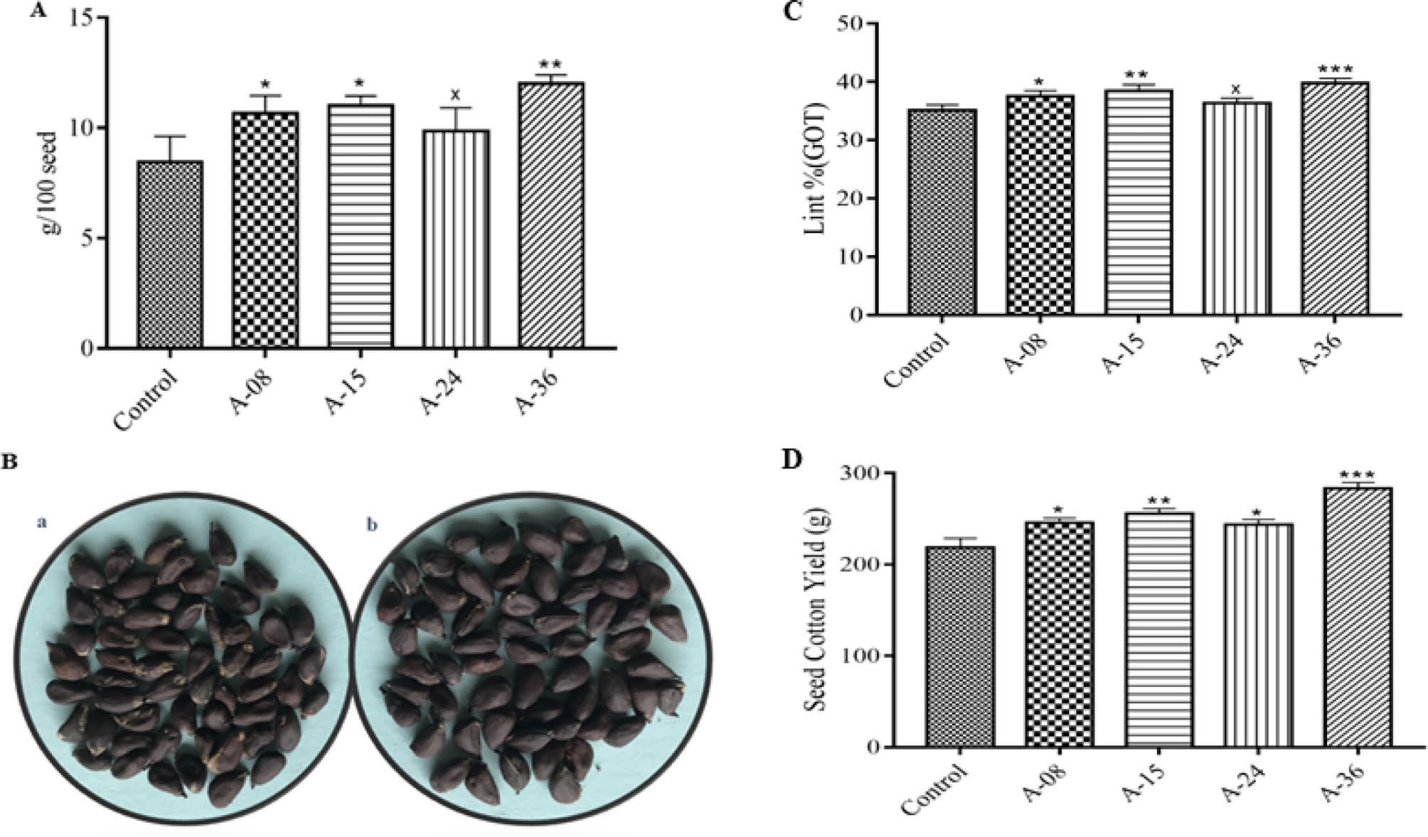
Seed Index Analysis and Pictorial representation (**A**) Average Seed index of transgenic and non-transgenic cotton lines (**B**) Pictorial representation of seed index (**a**) Non-transgenic cotton seeds (**b**) Transgenic cotton seeds (**C**) Lint percentage (GOT) (**D**) Seed cotton yield of transgenic and non-transgenic cotton lines. Each bar is the mean value representation of three replicates and One-way ANOVA was performed for statistical analysis.

The preferable expression of the *ACTIN1* gene in the embryo sac can also influence the seed weight. When the seed index of the transgenic cotton line in comparison to the non-transgenic control line was calculated by weighing 100 healthy disease-free seeds from each The seeds index was found to be 10.6, 11, 9.8, and 12.1 g sequentially in the transgenic cotton line A-08, A-15, A-24, and A-36 respectively while non-transgenic control cotton line the seed weight was recorded to be 8.4 g. Overall, a 1.2 to 3.7 g increment in seed index was recorded (Fig. 9A). Figure 9B(a,b) is a pictorial representation of the seed index of non-transgenic control and transgenic seeds. Ginning out turn (GOT) or lint percentage (lint %) of transgenic and control cotton lines was calculated using the lint-to-seed weight ratio. The lint percentage of transgenic cotton plants lines A-08, A-15, A-24, and A-36 was found to be 37.5, 38.4, 36.3, and 39.7% while the control line showed 35.1% of GOT. Three transgenic cotton lines, namely A-08, A-15, and A-36, significantly improved their lint percentage compared to the control cotton line (Fig. 9C). Seed cotton yield is an important parameter. The increase in seed cotton yield of 24.9 to 64.6 g was calculated in transgenic cotton lines compared to 218.8 g of the control line. The transgenic cotton line A-08, A-15, A-24, and A-36 were found to have 245.7, 255.5, 243.7, and 283.4 g of seed cotton yield respectively, while in the control cotton line, the seed cotton yield remained to be 218.8 g (Fig. 9D).

#### Fresh and dry cotton bolls weight

**Figure 10:**
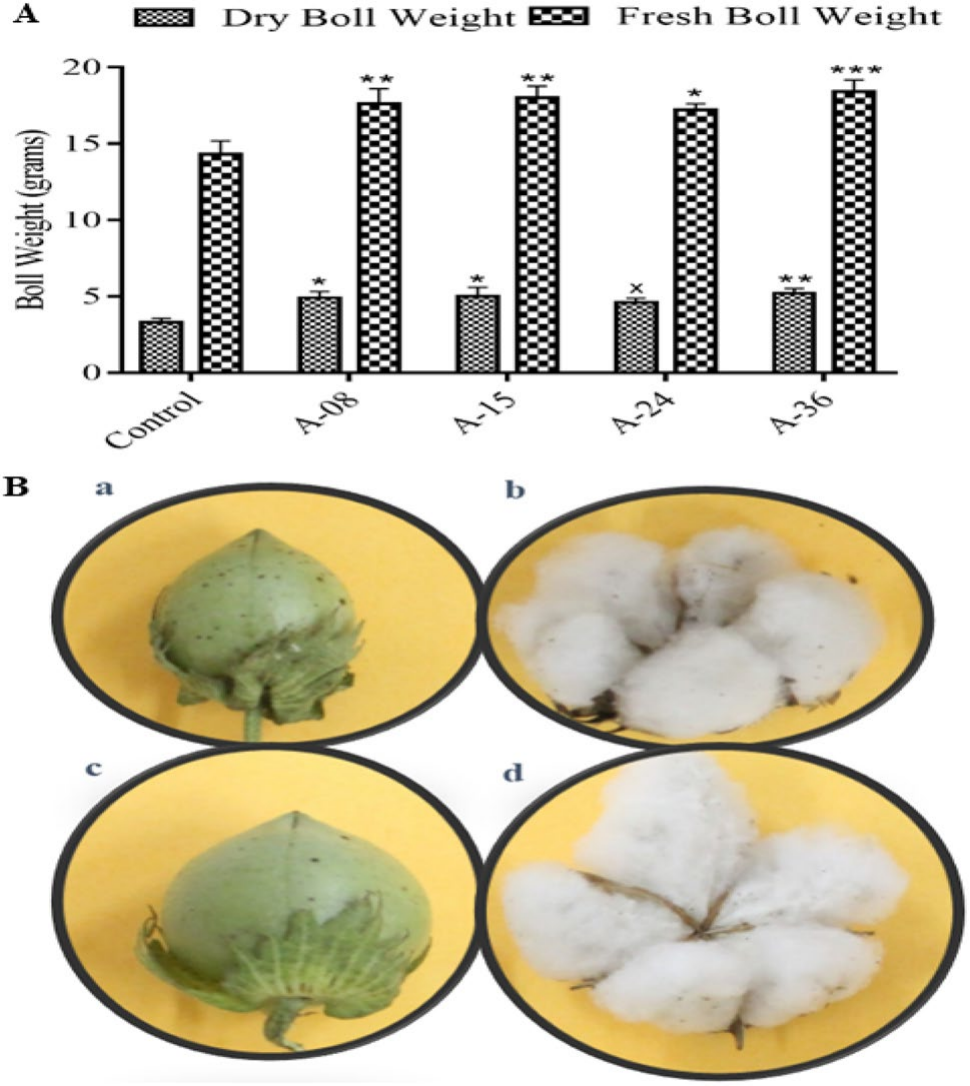
Fresh and Dry boll weight Analysis and Pictorial Representation (**A**) Fresh and Dry boll weight of transgenic and non-transgenic cotton lines. Each bar is the mean value representation calculated as described in Sect. 3.14.5. Two-way ANOVA was performed for statistical analysis (**B**) Pictorial representation of Fresh and Dry boll weight (**a**, **b**) Fresh and dry cotton bolls of non-transgenic control plant (**c**, **d**) Fresh and dry cotton bolls of transgenic plant.

The fresh and dry boll weight analysis of transgenic cotton plants showed an increase in average weight as compared to non-transgenic control cotton plants. Fresh weight was 17.6, 18.0, 17.2, and 18.4 g in transgenic cotton lines A-08, A-15, A-24, and A-36 compared to 14.3 g in the control line. The dry boll weight of these transgenic cotton lines was recorded to be 4.9, 5, 4.6, and 5.2 g in contrast to 3.3 g of the non-transgenic control line. A maximum of 28.6% and 57.5% increment in fresh and dry boll weight was recorded, respectively, in transgenic cotton lines (Fig. 10A). Figure 10B(a–d) represents fresh and dry cotton bolls taken from transgenic and non-transgenic control plants.

## Discussion

The expression of the *GhACTIN1* gene in cotton variety CEMB-88 was orchestrated through stable *Agrobacterium*-mediated transformation of the chemically synthesized codon-optimized *GhACTIN1* gene under the control of fiber-specific promoter, *GhSCFP,* cloned in pCAMBIA-1301, a plant expression vector, with *Pst1* and *Sac1* restriction sites (Fig. 1), as was done by Iqbal et al., Latif et al., and Rao et al.^15,16,18^. The transformation efficiency was calculated to be 1.04% and the results were consistent with transformation efficiencies reported by Rao et al. and Bajwa et al.^16,24^. Putative transgenic cotton plants were subjected to PCR analysis to confirm successful gene transformation and amplification of 577 bp DNA fragment (Fig. 3A) confirmed the presence of transgene as was reported by Puspito et al. and Iqbal et al.^25,26^. Transient expression through GUS assay from the cotton fiber of transgenic and non-transgenic control plants further helps to screen out transgenic cotton plants expressing the GUS marker gene. The appearance of blue color in fiber of transgenic cotton plants and complete absence of blue color in non-transgenic control cotton fiber (Fig. 3B) similar results were reported by Satyavathi et al. and Ahmed et al.^18,27^. The qRT-PCR analysis in advance generation (T_1_) at different fiber developmental stages such as initiation (4DPA), elongation (15DPA), and secondary wall synthesis (25DPA) shows that *GhACTIN1* gene expression reaches its maximum at the fiber elongation phase which is an indication that transgene has less expression in fiber initiation and secondary cell-wall synthesis phases (Fig. 4) the results are parallel with the study of Li et al.^3^ who reported major role of *GhACTIN1* in fiber elongation phase but not in the initiation and secondary wall synthesis.

Fluorescent In situ Hybridization analysis is an efficient way to locate the integrated exogenous gene and determine the copy number in transgenic cotton plants^28^. The T_2_ generation transgenic cotton plant line A-36 was selected to locate *GhACTIN1* gene integration and copy numbers at the chromosome level. Transgenic cotton line A-36 was selected for FISH analysis owing to its much-improved fiber characteristics and higher transgene expression. The single bright fluorescent signal on chromosome number 8 indicates that there was a single copy number in hemizygous form (Fig. 5A,B). Puspito et al.^25^ also reported similar results in transgenic cotton plants harboring the insect and weedicide-resistant genes by using the same gene transformation procedure, which reflects the procedural capacity for the introduction of the least transgene copy number which is preferred for higher expression. Over-expression of many actin-binding proteins results in actin bundling and an increased quantity of F-actin filaments^13,29^. During developing cotton fiber elongation stage, quantification of F-actin filament in *GhACTIN1* over-expressed transgenic cotton plants was found to be increased by up to 8.7% (Fig. 6A). F-actin filaments are reported to be important in the regulation of fiber length, strength, and maturation^3,13^. Actin microtubules result in the deposition of cellulose fibrils in the cell membrane and cell wall by delivering and positioning activated cellulose synthase complexes in Arabidopsis^30^. Consistent with this study, up to 4.7% higher contents of cellulose in fibers of transgenic cotton lines were recorded by over-expression of the *GhACTIN1* gene (Fig. 6B).

The impact of over-expression of *GhACTIN1* on fiber strength and length displayed significant improvements, up to 24.2% in fiber strength and 5.3% in fiber length, compared to the non-transgenic control cotton line (Fig. 7A,E). The results were consistent with the study of Li et al.^3^, where downregulation of *GhACTIN1* resulted in disruption of actin cytoskeleton in cotton fibers, and consequently, reduced the fiber length and strength. Increment in transgenic cotton fiber length can also correlate with increased cellulose contents in transgenic cotton lines (Fig. 6B). Higher cellulose contents result in higher turgor pressure, and hence it helps in cotton fiber elongation^31^. We have also found that over-expression of *the GhACTIN1* gene has more impact on fiber strength than fiber length. This is because higher expression of *GhACTIN1* during the fiber elongation phase turned out in the higher quantity of F-actin in transgenic cotton fibers compared to the non-transgenic control. Thicker F-actin bundles pre-terminate elongation phase and pre-start secondary cell-wall synthesis. This higher quantity of F-actin during the elongation stage reorients the F-actin filament bundles from transverse to oblique position, and F-actin abundance results in higher strength in cotton fiber^13^. Furthermore, SEM fibers analysis of transgenic cotton lines (Fig. 7F) exhibited higher twists per unit area which can be correlated to increased fiber strength as enhanced cellulose deposition at the cell wall peripheral region leads to enhanced fiber strength and fineness^32,33^. Micronaire (combination of fiber fineness and maturity) values and maturity ratio were also significantly improved up to 24.3% and 10.5%, respectively, in transgenic cotton lines (Figs. 8A, 6C). Moreover, SEM analysis (Fig. 8B) illustrates the fiber of transgenic and non-transgenic control cotton lines where increased smoothness of fiber taken from transgenic cotton lines is evident as compared to fiber of non-transgenic cotton lines Bajwa et al.^24^ and Li et al.^34^ have reported the improvement in micronaire values and maturity ratio through *GhEXPA8* and *GhUGP-1* genes introduction into cotton plants. Although the maximum uniformity index of cotton fiber was recorded to be 85.6% in transgenic cotton lines, however, no significant improvement in the overall uniformity index was recorded when compared to cotton fiber of the non-transgenic control line (Fig. 6D).

Agronomical characteristics of transgenic cotton plants in comparison to the non-transgenic control line were taken into account to define any comparable change in both groups of plants which may be attributed to insertional change or any contribution from genetic modification as was done by Shu et al.^35^, where they found out change in agronomical as well as morphological traits such as plant height, grain-size, seed-fertility in transgenic rice carrying *Cry1Ab/CryaAc* gene. The seed index is an important factor considering the effect of transgene on seed weight. A total of 1.2 to 3.7 g weight of seed with a maximum increase of up to 44.04% was recorded in transgenic cotton lines (Fig. 9A). These results can be correlated with the fact that the *ACTIN1* gene is expressed in the embryo sac and ovules^36,37^. The increase in average fresh and dry boll weight can directly correlate with the high seed index, seed cotton yield, and lint percentage (GOT). A maximum 28.6% and 57.5% increment in fresh and dry boll weight were also recorded respectively in the transgenic cotton plants as compared to non-transgenic control (Fig. 10A) and up to 4.6% increment in lint percentage (GOT) while a 29.5% increment in seed cotton yield was also observed (Fig. 9C,D) as compared to non-transgenic cotton line. Similar results were reported by Khan et al.^38^ and Usman et al.^43^.

The transpiration rate of transgenic cotton plants was found to be in a positive correlation with the photosynthetic rate, while no significant effect was found on the water use efficiency of the transgenic cotton line except for the A-36 transgenic line, which could be attributed to an environmental factor or individual plant response.^39^ Hillel and Hatfield^45^ have reported that different physiological factors such as stomatal conductance, photosynthetic rate, water use efficiency, and transpiration rate were positively correlated.

## Conclusion

The study was intended to characterize the *GhACTIN1* gene under the control of the *GhSCFP* promoter for fiber trait improvement. The results obtained by over-expression of the transgene were very promising and found to have a positive impact on fiber length, strength, micronaire, and maturity however, molecular, microscopic, and fiber analyses suggest that the *GhACTIN1* gene is more associated with fiber strength and micronaire (fineness and maturity) than fiber length. Physiological analyses revealed that the *GhACTIN1* gene also has an impact on plant physiology which in turn resulted in the improvement of plant agronomical traits such as seed cotton yield and lint percentage. Cotton fiber is a multigeneic trait, so using biotechnological approaches different desirable fiber traits can be brought together to meet the demand of the textile industry. This study will contribute to this main goal.

### Supplementary Information

Supplementary Information 1.

Supplementary Information 2.

## Data Availability

The data generated or collected during research has been given in the manuscript and supplementary file.
