# Supplementary figures and images for: Over-expression of GhACTIN1 under the control of GhSCFP promoter improves cotton fiber and yield
Source: Sci Rep. 2023 Oct 26;13:18377. doi: 10.1038/s41598-023-45782-0 (PMC10603119; doi:10.1038/s41598-023-45782-0)

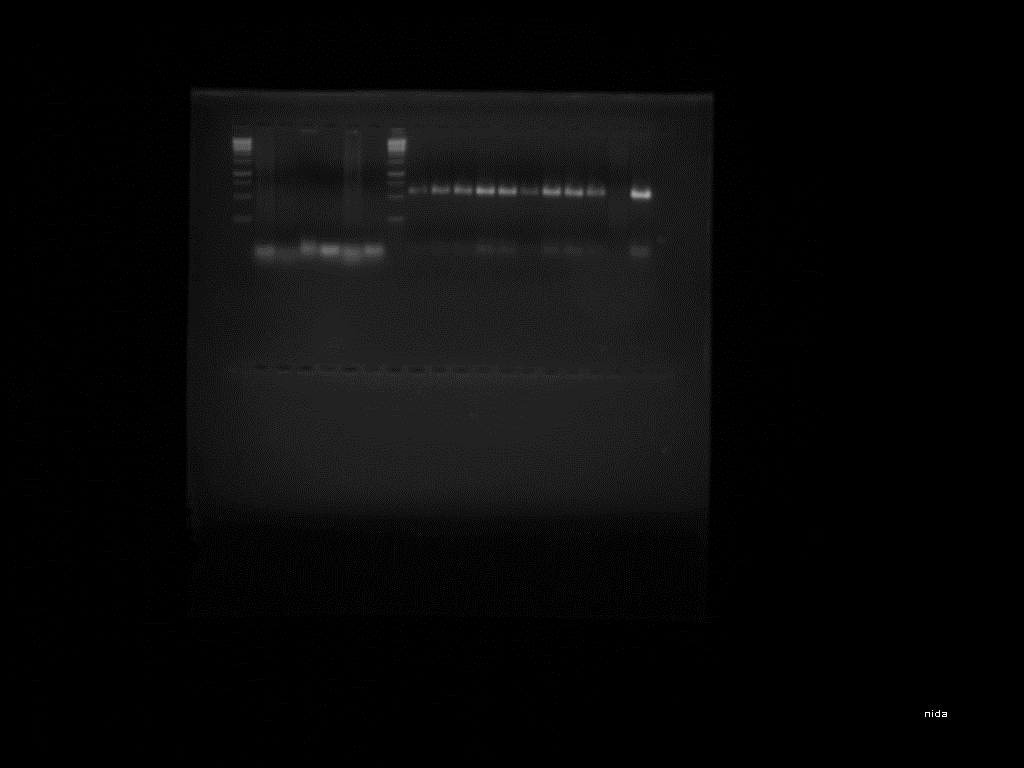


Figure 3A in manuscript.


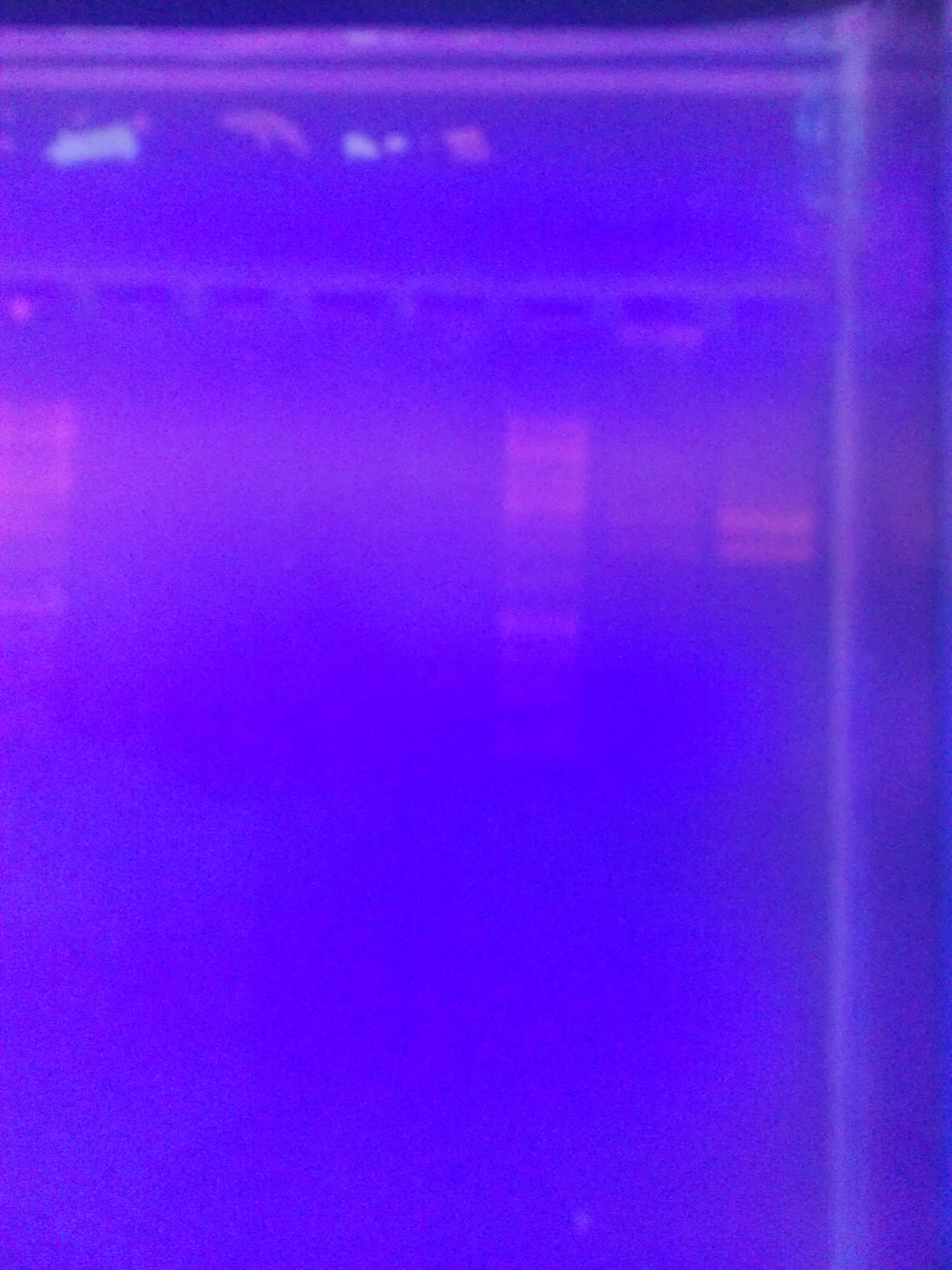


SFigure1.


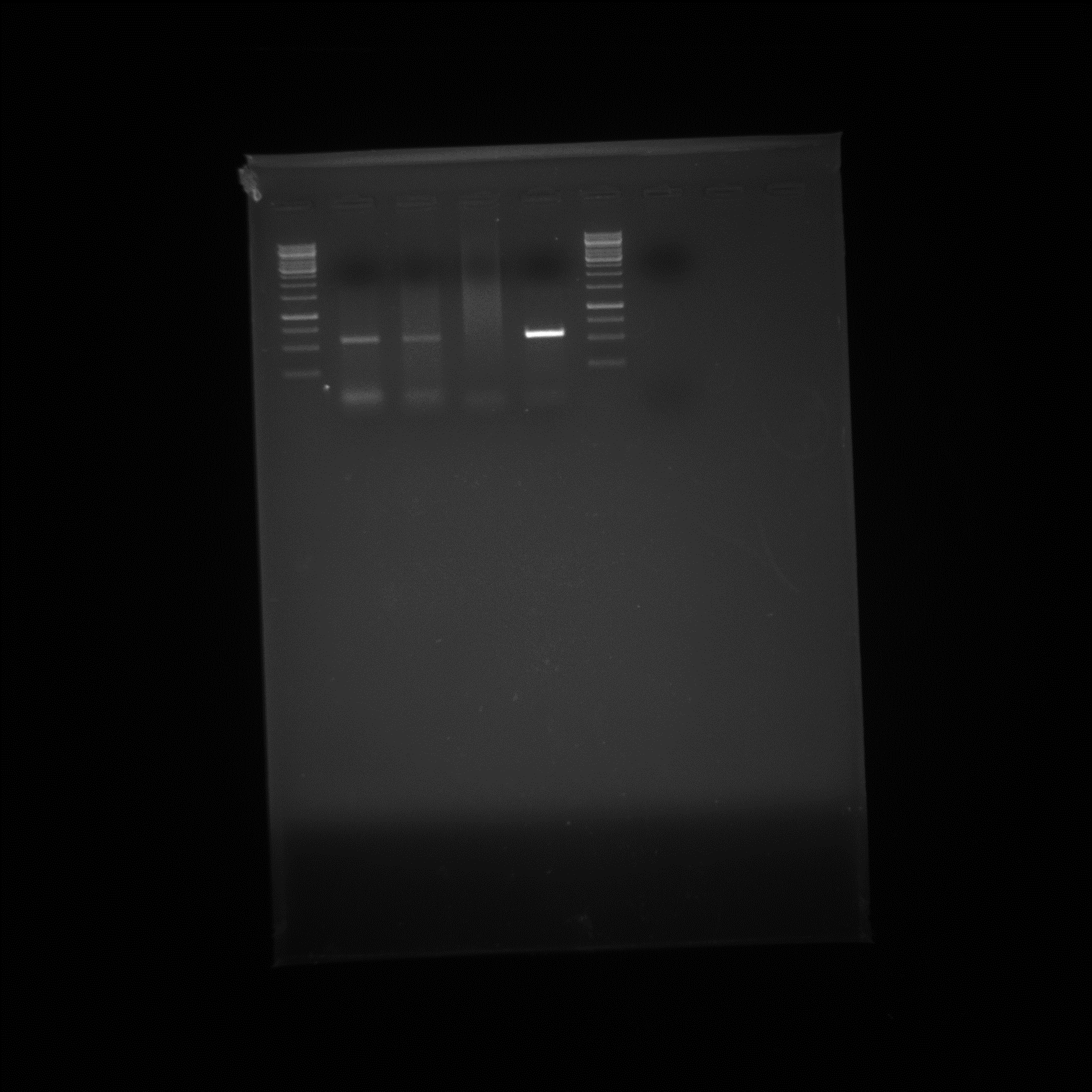


SFigure2A.


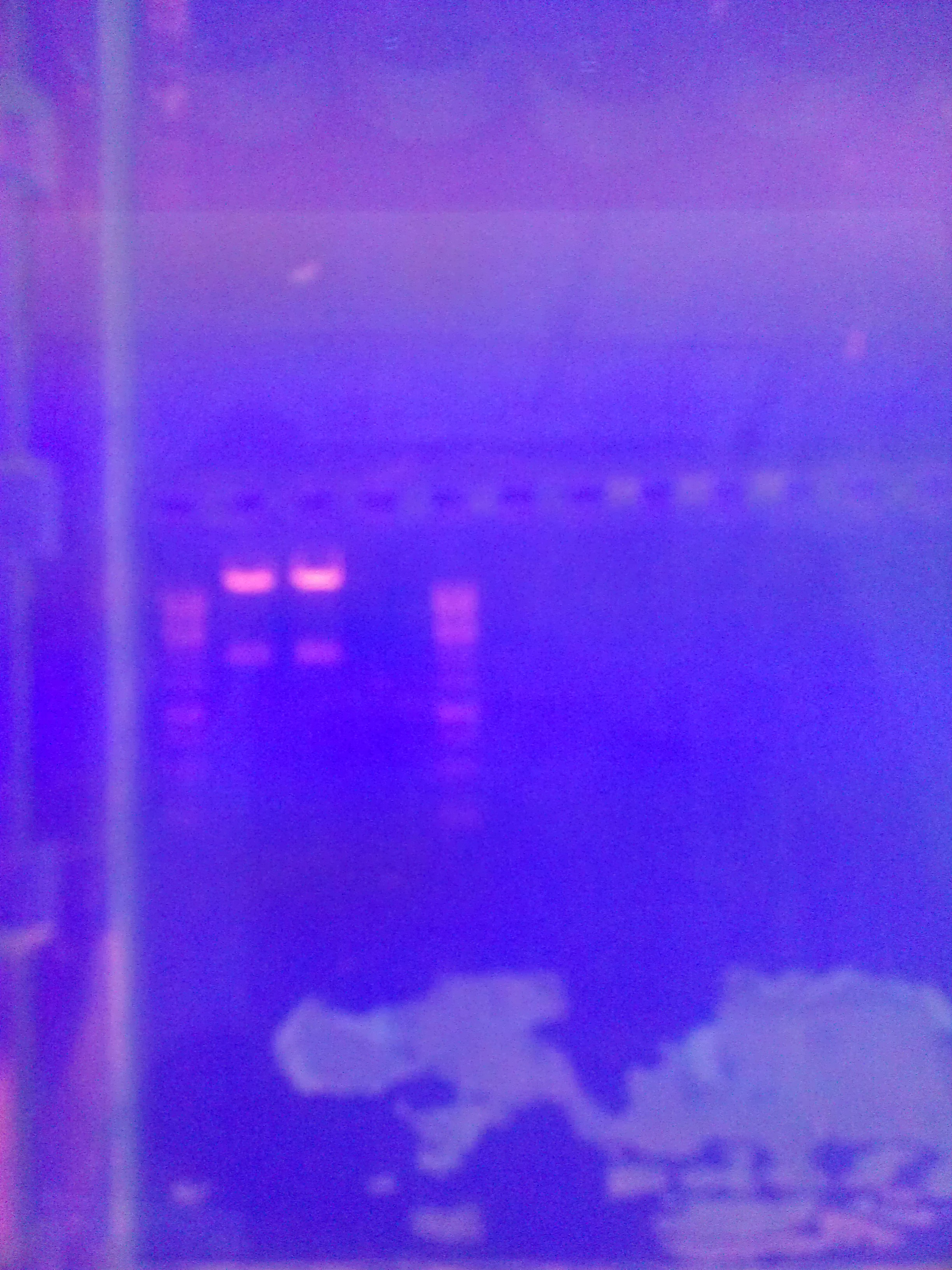


SFigure2 B


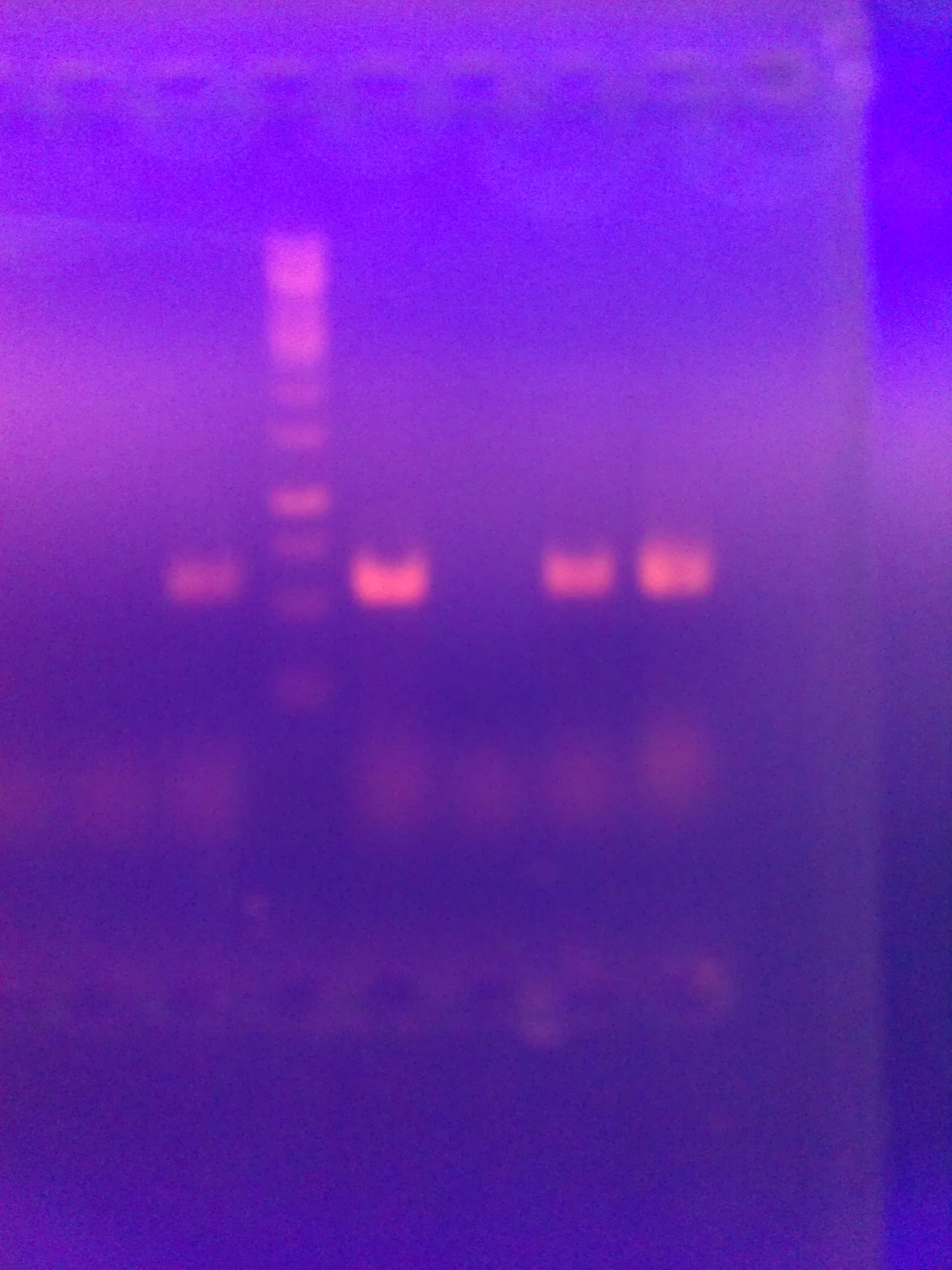


SFigure3.


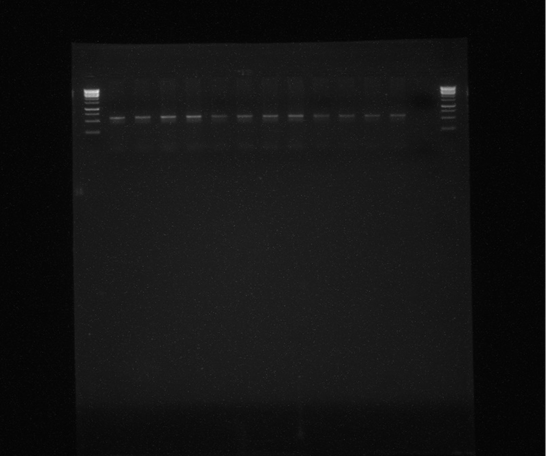


SFigure5.

Supplement: Supplementary file 1 — Supplementary Information 1. [file 41598_2023_45782_MOESM1_ESM.docx]
