# Supplementary material for: Over-expression of GhACTIN1 under the control of GhSCFP promoter improves cotton fiber and yield
Source: Sci Rep. 2023 Oct 26;13:18377. doi: 10.1038/s41598-023-45782-0 (PMC10603119; doi:10.1038/s41598-023-45782-0)
Supplement: Supplementary file 2 — Supplementary Information 2. [file 41598_2023_45782_MOESM2_ESM.docx]

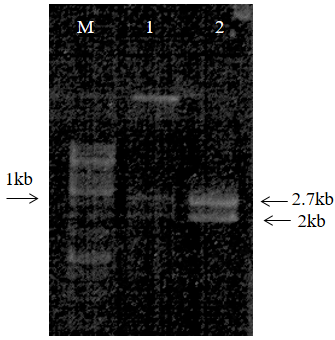


SFigure 1: Confirmation of pUC57_GhACTIN1 Cloning through Restriction Digestion Analyses. M: 1kb Ladder; Lane 2 and 3: 2kb fragment of GhACTIN1 cassette digested with pst1 and Sac1 from pUC57_GhACTIN1 plasmid while 2,7kb is released vector


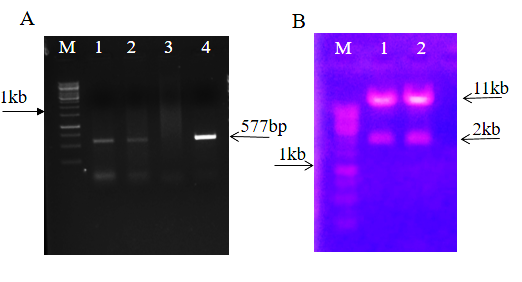


SFigure2: Confirmation of pCAMBIA-1301_GhACTIN1 Construct by PCR Amplification and Restriction Digestion Analysis. **(A)** M: 1Kb Ladder; Lane 1 and 2: Amplified Colonies; Lane 3: Unamplified Negative Control (Non-transformed Colony); Lane 4: Positive Control (pUC57_GhActin) **(B)** Restriction Digestion, M: 1kb Ladder, Lane 1 and 2: 2kb fragment of GhACTIN1 cassette digested with pst1 and Sac1 and 11kb vector from pCAMBIA-1301_GhACTIN1 plasmid


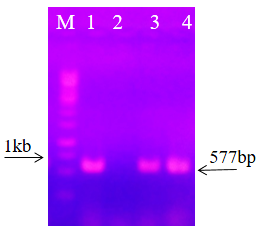


SFigure3: Confirmation of GhACTIN1_pCAMBIA-1301 plasmid transformation in A. tumefaciens through Colony PCR. M, 1kb Ladder; Lane 1 Positive control (pCAMBIA-1301_GhACTIN1 Plasmid), Lane 2 Negative Control (Non-transformed colony), Lane 3 and 4 Amplified Colonies


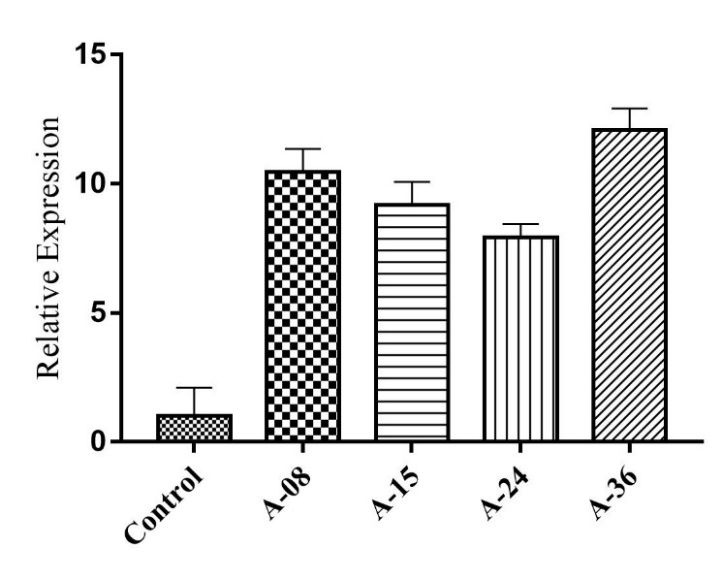


SFigure 4: Quantitative Real-time mRNA Expression of GhACTIN1 gene. Each bar is the mean value representation of three replicates


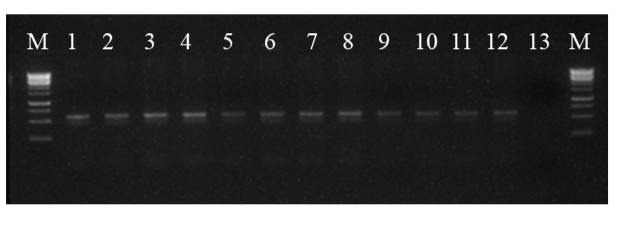


SFigure 5: PCR Analysis of Transgenic Cotton plants of T1 Progeny. M: 1kb Ladder; Lane 1-11 Transgenic cotton plants with an amplification of 577bp fragment; Lane 12 Positive Control plant from T0; Lane 13 Negative Control (Non-transgenic)
